# Complete genome sequence of *Luteolibacter* sp. strain LG18, an L-glucose-utilizing bacterium isolated from soil

**DOI:** 10.1128/mra.00888-23

**Published:** 2024-02-14

**Authors:** Masashi Yachida, Akira Nakamura

**Affiliations:** 1 Institute of Life and Environmental Sciences, University of Tsukuba, Tsukuba, Japan; 2 Microbiology Research Center for Sustainability (MiCS), University of Tsukuba, Tsukuba, Japan; University of Maryland School of Medicine, Baltimore, Maryland, USA

**Keywords:** L-glucose utilization, *Luteolibacter* sp., complete genome sequence

## Abstract

An L-glucose-utilizing bacterium, *Luteolibacter* sp. strain LG18, was isolated from soil, and the complete genome sequence was determined. Strain LG18 contained a single circular chromosome of 5.80 Mb with a G + C content of 64.5%, in which 4,598 protein-coding genes, 9 rRNA, and 56 tRNA genes were identified.

## ANNOUNCEMENT

**Fig 1:**
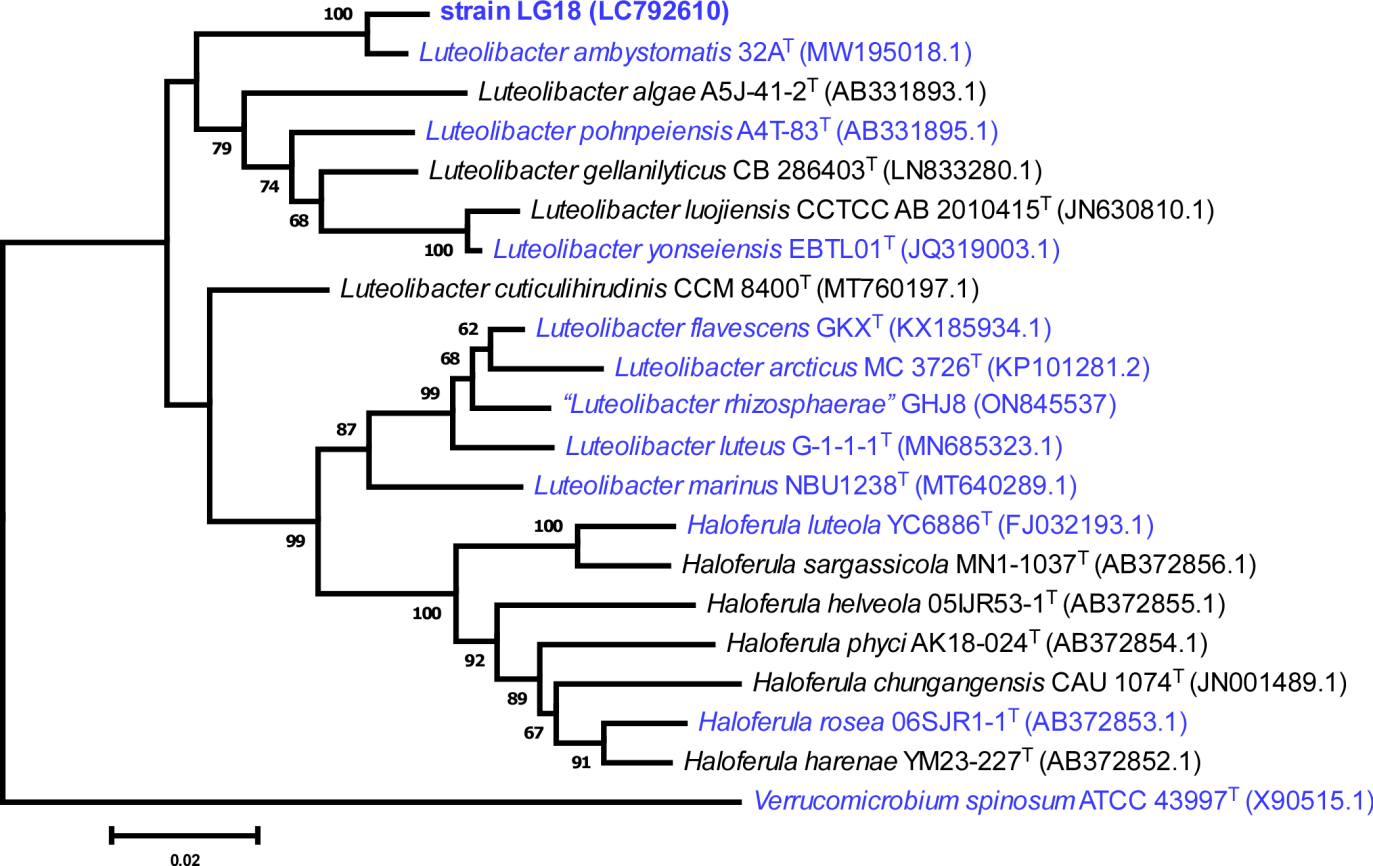
Phylogenetic tree based on the 16S rRNA gene sequence of strain LG18 and related taxa. The tree was constructed by the neighbor-joining method in MEGA11. Accession numbers are shown in parentheses. The organisms whose genome sequences are available are shown in blue.

Previously, we reported isolation of an L-glucose-utilizing bacterium, *Paracoccus laeviglucosivorans* (1), classified into the phylum *Pseudomonadota*. Since then, no reports are present on L-glucose-utilizing microorganisms. Our continuing screening has led us to isolate strain LG18 from a garden soil of a private house in Japan (35.578917 N 139.350222 E) by enrichment cultivation in a minimal medium containing L-glucose (2) at 28°C for 4 days with shaking and colony formation on plates of the same medium at 30°C for 4 days. Strain LG18 grew at 30°C on the minimal medium plates containing D-glucose as well as L-glucose, and R2A broth. The 16S rRNA gene sequence, determined using the DNA fragment obtained by colony PCR with primers 27F (5′-AGAGTTTGATCMTGGCTCAG-3′) and 1492R (5′-GGTTACCTTGTTACGACTT-3′), and a phylogenetic analysis by the neighbor-joining method demonstrated that strain LG18 belonged to the genus *Luteolibacter* of the phylum *Verrucomicrobiota*, with *L. ambystomatis* as the closest related species (Fig. 1).

Genomic DNA was isolated from a culture grown in R2A broth at 28°C for 3 days with a Wizard genomic DNA purification kit (Promega). Short-read and long-read sequencing were performed using MiSeq (Illumina) and GridION [Oxford Nanopore Technologies (ONT)] at Fasmac (Japan) and Bioengineering Laboratory (Japan), respectively, using different preparations with the same procedure. For short-read sequencing, genomic DNA (300 ng) was used for fragmentation and construction of a library with a KAPA HyperPlus Library Preparation Kit (Roche) without PCR amplification, according to manufacturer’s protocol, yielding a library with an average length of 933 bp. Paired-end (2 × 300 bp) sequencing was performed and ~1.78 million paired-end raw reads were obtained. The data were processed using Cutadapt v. 2.7 (3) to trim adapters, and low-quality reads (Q < 20; read length, <127 bases) were removed by Sickle v. 1.33 (4). For long-read sequencing, Native Barcoding Expansion 13–24 (ONT) and Ligation Sequencing Kit (ONT) were used to construct a library with barcodes, using 1 µg of the genomic DNA. Then the library was analyzed on a GridION apparatus equipped with an R9.4.1 flow cell (ONT). Base calling was performed with Guppy v. 4.0.11 (ONT) to generate 134,234 reads with an average length of 13,279 bp. Trimming of adapters and short reads (≤1,000 bases) were conducted with Porechop v. 0.2.3 (5) and Filtlong v. 0.2.0 (https://github.com/rrwick/Filtlong), respectively, and the remaining 119,258 reads were used for the hybrid assembly.

The long- and short-read data were assembled *de novo* with Unicycler v. 0.4.7 (6), and the assembled genome data were checked by CheckM v. 1.1.2 (7) with the default settings, resulting in the genome coverage of ×488. Strain LG18 contained a single circular genome of 5,803,342 bp with a G + C content of 64.5%. Automatic annotation by DFAST v. 1.4.0 (8) revealed that the chromosome contained 4,598 protein-coding genes, 9 rRNA genes, and 56 tRNA genes.

The complete genome sequence of this strain will support a study for L-glucose catabolism of this strain and future studies of the genus *Luteolibacter*.

## Data Availability

The 16S rRNA gene sequence accession number for strain LG18 is LC792610. The complete genome sequence of *Luteolibacter* sp. strain LG18 is available from DDBJ/EMBL/GenBank with accession number AP024600. Raw sequence data were deposited in the DRA database under the accession numbers DRA011734 [DRR279230 (MiSeq) and DRR279231 (GridIon)] (BioProject number PRJDB11401 and BioSample number SAMD00289724).
